# Data, Signal and Image Processing and Applications in Sensors II

**DOI:** 10.3390/s24082555

**Published:** 2024-04-16

**Authors:** Manuel J. C. S. Reis

**Affiliations:** 1 Engineering Department, University of Trás-os-Montes e Alto Douro (UTAD), 5001-801 Vila Real, Portugal; mcabral@utad.pt; 2 Institute of Electronics and Informatics Engineering of Aveiro (IEETA), 3810-193 Aveiro, Portugal

A vast and ever-growing amount of data in various domains and modalities is readily available, being the rapid advance of sensor technology one of its main contributor. However, presenting raw signal data collected directly from sensors is sometimes inappropriate, due to the presence of, for example, noise or distortion, among others. In order to obtain relevant and insightful metrics from sensors signals’ data, further enhancement of the sensor signals acquired, such as the noise reduction in the one-dimensional electroencephalographic (EEG) signals or colour correction in the endoscopic images, and their analysis by computer-based medical systems, is needed. The processing of the data in itself and the consequent extraction of useful information are also vital and included in the topics of this Special Issue, being this an extension of the first special issue on this subject (https://www.mdpi.com/journal/sensors/special_issues/signal_sensors, accessed on 15 March 2024).

This second edition of this SI of Sensors aims to showcase progress in the advancement, assessment, and implementation of algorithms and methodologies for processing data, signals, and images across diverse sensor types and sensing approaches. Both empirical and theoretical findings, along with review articles, were taken into account.

The quantity of manuscripts submitted directly indicates the significant interest in this topic within the research community, with a total of 42 manuscripts received. Among these, 27 papers of high quality were accepted and published, while 15 papers were rejected. As customary, the Sensors journal upheld its standards by subjecting all submitted manuscripts to a thorough peer-review process.

In the forthcoming presentation, I will utilize the exact wording of the authors to effectively convey the contributions of each paper, as well as trying to provide the readers with a summary of each paper.

Pires et al., in contribution 1, discuss medicine’s evolution towards personalized care, focusing on cardiovascular diseases. They propose an AI-based system to empower patients via continuous monitoring and personalized treatment. The system aims to realize 5P (Predictive, Preventive, Participatory, Personalized, and Precision) medicine principles using data from wearables and smart devices. Key features include learning algorithms for data analysis, event prediction, alarm generation, and healthy behaviour promotion. It aims to boost patient engagement and contact with healthcare professionals. Cardiovascular diseases are highlighted as major causes of disability and death, emphasizing proactive management. Computational intelligence and device data integration enable efficient healthcare management, representing a comprehensive approach to disease management. It is positioned to enhance patient well-being and promote global public health.

Hao et al., in contribution 2, introduced a spectrum correction algorithm, decomposition filtering-based dual-window correction (DFBDWC), for improving target distance accuracy in frequency modulated continuous wave (FMCW) laser ranging. Traditional methods face challenges from white Gaussian noise (WGN), spectrum leakage, and the picket fence effect, yielding unsatisfactory results. DFBDWC employs decomposition filtering and a dual-window approach to effectively mitigate these issues. Experimental validation demonstrates its superior performance compared to traditional methods discrete Fourier transform algorithm, phase demodulation, enhanced cross-correlation, Ratio, Chirp Z-transform, and enhanced cross-correlation algorithm. DFBDWC significantly reduces the maximum error from 0.7937 m to 0.0407 m, improving accuracy and frequency resolution while minimizing the impact of noise and spectrum leakage. Accurate WGN estimation and effective filtering contribute to its success. Moreover, a double Hann window reduces spectrum leakage, while utilizing two main spectral lines enhances overall performance.

Gerasimova et al., in contribution 3, propose a novel memristive interface composed of two FitzHugh–Nagumo electronic neurons connected via a metal–oxide memristive synaptic device. A hardware–software complex based on a commercial data acquisition system is developed to record signals from a presynaptic neuron and transmit them to a postsynaptic neuron through the memristive device. Both numerical simulations and experiments demonstrate complex dynamics, including chaos and various types of neural synchronization. The system offers simplicity and real-time performance, with the amplitude of the presynaptic signal leading to potentiation of the memristive device and adaptive modulation of the postsynaptic neuron output. Due to its stochastic nature, the memristive interface simulates real synaptic connections, holding promise for neuro-prosthetic applications. The authors investigate the dynamics of two coupled FitzHugh–Nagumo neuron generators through a metal–oxide memristive device, showcasing stochastic plasticity and various synchronous regimes. The relative compactness and high sensitivity of the proposed neuro-memristive device make it promising for applications in bio-robotics and bioengineering.

Fiedler et al., in contribution 4, introduce Simultaneous Face and Person Detection (SFPD), aiming for real-time detection of faces and persons. Combining these tasks is essential for computer vision applications like face recognition and human–robot interaction. SFPD employs multi-task learning, addressing the lack of datasets with both annotations algorithmically. It utilizes a joint convolutional neural network backbone with shared feature maps and separate detection layers for each task. SFPD doesn’t need auxiliary steps during training, such as pre-training individual network parts or additional annotations. Evaluation shows SFPD’s effectiveness in detection performance and speed, achieving 40 frames per second. Comparative analysis demonstrates its superiority in processing speed, detection performance, or providing both face and person detections. Overall, SFPD offers a valuable real-time framework for various applications, especially in human–robot interaction scenarios.

Fuentes et al., in contribution 5, tackle real-time IoT data visualization without costly hardware. They propose an augmented reality (AR)-based solution using consumer-grade smartphones. The system enables real-time data visualization from IoT devices via AR, with added security. Tests confirm the solution’s effectiveness in accessing IoT data, smartphone-device interactions, and identifying optimized AR markers. Results show the feasibility of using smartphones for IoT device management in diverse environments. Key contributions include an architecture for simplified AR IoT data visualization and a functional prototype validation. Future work may explore an AR marker generator for improved performance and usability.

Fuentes et al., in contribution 6, tackle Portugal’s aging population issues, especially in rural areas where elders often face isolation and resource constraints. They propose an affordable Ambient Assisted Living (AAL) system to monitor elderly activities at home, respecting their privacy. Using low-cost IoT sensors and computer vision, the system recognizes elderly activities and was successfully tested in a simulated scenario. It enables remote caregiving, allowing independent living with assistance when needed, while ensuring privacy. The prototype, utilizing Raspberry Pi Zero W, effectively monitors specific home areas. Future enhancements may optimize Raspberry Pi processing, incorporate grey areas to reduce false positives, and introduce automatic alerts for caregivers. Overall, this solution promises to support independent living for elders, enhancing safety and well-being.

Pilyugina et al., in contribution 7, conducted a study to find effective feature extraction methods from auditory steady-state responses (ASSR) data to differentiate between auditory octave illusion and non-illusion groups. Various feature selection techniques, including univariate selection, recursive feature elimination, principal component analysis, and feature importance, were compared. Machine learning algorithms such as linear regression, random forest, and support vector machine (SVM) were employed to evaluate these methods. The study revealed that combining univariate selection with SVM achieved the highest accuracy of 75%, surpassing the 66.6% accuracy obtained without feature selection. These findings provide a foundation for further research into understanding the mechanism behind the octave illusion phenomenon and developing automatic classification algorithms for octave illusions.

Motor imagery (MI)-based brain–computer interfaces (BCIs) are crucial for device control via brain activity. Despite this, complex inter-communication among brain regions during motor tasks presents challenges for isolating relevant neural patterns. To tackle this, Awais et al., in contribution 8, utilized effective brain connectivity measures like partial directed coherence (PDC) to capture inter-channel/region relationships during motor imagination. Statistical analysis identified significant connectivity pairs, and four classification algorithms (SVM, KNN, decision tree, and probabilistic neural network) predicted MI patterns using PhysioNet EEG data. Results showed the probabilistic neural network (PNN) classifier with PDC features achieved 98.65% accuracy, highlighting PDC’s superiority over DTF in classification. Leveraging brain connectivity enhances neural pattern understanding, advancing BCI applications. Future research might explore graph theory and optimization for improved real-time BCI applications, especially for those with motor disabilities.

Bas-Calopa et al., in contribution 9, investigate the impact of low-pressure environments and high-operating frequencies on visual corona discharges, crucial for understanding arc tracking and insulation degradation in aircraft wiring systems as more electric and all-electric aircraft become prevalent. Experimentation employs a rod-to-plane electrode setup across pressure (20–100 kPa) and frequency (50–1000 Hz) ranges relevant to aircraft applications. A low-cost, high-resolution CMOS imaging sensor is utilized for corona detection, offering simplicity and sensitivity, while leakage current analysis serves as a complementary method. Results reveal that corona extinction voltage (CEV) increases notably with air pressure, while frequency exhibits a lesser effect, causing CEV to decrease within certain pressure ranges. The CMOS sensor demonstrates sufficient sensitivity for corona detection in low-pressure environments across various frequencies, offering potential for insulation system design in modern aircraft. Additionally, the study underscores the comparable sensitivity between the CMOS sensor and leakage current analysis, with minor discrepancies diminishing at higher frequencies.

Ebrahimi et al., in contribution 10, investigate the effectiveness of Robust Principal Component Analysis (PCA) matrix decomposition alongside advanced methods (PCT, PPT, and PLST) for analyzing pulsed thermography thermal data in carbon fibre-reinforced polymer (CFRP) materials. Using an academic sample with artificial defects, they assess defect detection and segmentation using CNR and similarity coefficient. Results show significant CNR improvements with Robust PCA pre-processing, enhancing defect detectability by up to 164%, 237%, and 80% for different defect types. Pre-processing notably improves CNR for FBHs and POs, with enhancements ranging from 0.43% to 115.88% and from 13.48% to 216.63%, respectively. Postprocessing enhances results for FBHs and POs by 9.62% to 296.9% and 16.98% to 92.6%, respectively. Robust PCA enhances defect detectability for PCT, PPT, and PLST methods, surpassing PLST for 69% of defects. Pre-processing enhances segmentation potential for all methods, with PLST showing improvements for both pre- and post-processing. The study concludes that Robust PCA pre-processing substantially enhances anomaly detection in pulsed thermography for CFRP materials, with implications for NDT. Further research should extend these techniques to diverse materials for enhanced practicality in NDT.

Jang et al., in contribution 11, presented a new method for detecting vital signals using multiple radar systems to reduce signal degradation from body movement. By analyzing phase variation in continuous-wave radar signals caused by respiration and heartbeat, the method employs two adjacent radars with different lines-of-sight to capture correlated signals, enhancing differences in organ movement asymmetry. Operating at different frequencies within the 5.8 GHz band and strategically positioned, the radars improve signal-to-noise ratio during vital signal detection. Experimental results showed 97.8% accuracy in vital signal detection, even with subjects moving at velocities up to 53.4 mm/s. The configuration and signal processing method effectively utilize asymmetrical organ movements, improving signal-to-noise ratio and detection accuracy, especially during body movement. Extensive testing demonstrated noise reduction in the low-frequency range and significant enhancements in signal-to-noise ratio and detection accuracy across various radar angles. Overall, this method offers robust vital signal detection, even in dynamic environments with substantial body movement.

Maddirala et al., in contribution 12, tackle the issue of eliminating eye-blink artifacts from single-channel electroencephalogram (EEG) signals, often recorded using portable EEG devices. These artifacts, stemming from eyelid blinking or eye movements, distort EEG measurements, impacting brain activity interpretation. Traditional artifact removal methods are inadequate for single-channel EEG signals, necessitating novel techniques. The proposed approach combines singular spectrum analysis with continuous wavelet transform and k-means clustering to remove eye-blink artifacts while retaining low-frequency EEG data. Assessment on synthetic and real EEG datasets validates the method’s superiority over existing techniques. Focused on pre-frontal channel EEG signals, it holds promise for online applications employing such channels. The study highlights successful artifact removal without sacrificing original EEG information, suggesting potential in real-time EEG monitoring and classification tasks. Future research should examine the method’s performance in classification scenarios, anticipating favourable outcomes based on demonstrated artifact removal effectiveness.

Murtiyoso et al. in contribution 13, proposed integrating AI-based semantic segmentation into photogrammetric workflows to automate semantically classified point cloud creation. Leveraging deep learning and semantic segmentation advancements, the method uses pretrained neural networks for automatic image masking and dense image matching. By starting with semantic classification in the photogrammetric process, the workflow is streamlined to generate labelled point clouds. Results demonstrate process automation feasibility, with promising assessments for specific classes like building facades and windows. Emphasizing the advantage of abundant 2D image label data for neural network training, challenges remain in handling underrepresented classes and optimizing training data generation. Future research should explore semantic photogrammetry in various settings, refining training data methods for close-range photogrammetry. Overall, the study provides a proof of concept for AI integration into photogrammetric tasks, setting the stage for semantic photogrammetry advancement.

Mohanna et al., in contribution 14, introduced a method to tackle radar shadow effects in FMCW radars, which hinder target discrimination when one target is in another’s shadow region. Utilizing CNNs on spectrograms from STFT analysis, the method determines if a target is in another’s shadow. Achieving 92% test accuracy with a 2.86% standard deviation, it effectively discerns scenarios with one or two targets. Using MobileNet architecture pretrained on Imagenet, the model attains high accuracy with low parameters, suitable for real-time use. Future research should test the solution on hardware like Raspberry Pi and extend it for tracking multiple moving targets in cluttered settings. While supervised learning is effective, an unsupervised approach may be needed for scenarios with unpredictable classes. Overall, the method offers a promising solution for mitigating radar shadow effects in FMCW radar, with diverse target detection and tracking applications.

Manian et al., in contribution 15, propose a semi-supervised method for labelling and classifying hyperspectral images, addressing the challenge of acquiring ground-truth data, which is time-consuming and resource-intensive. The method comprises two stages: unsupervised and supervised. In the unsupervised stage, image enhancement and clustering generate ground-truth data. The supervised stage involves pre-processing, feature extraction, and ensemble learning using various machine learning models. The ensemble method achieves high accuracy, with gradient boosting performing the best. It’s effective for classifying Lake Erie and Jasper hyperspectral datasets, achieving accuracy rates of 100% and 93.74%, respectively. Additionally, it efficiently detects cloud pixels and water pollutants, useful for environmental monitoring. The choice of normalization scheme and number of PCA bands significantly impacts model performance and efficiency. The method runs significantly faster on cloud servers, making it practical for large-scale image processing tasks. Overall, the semi-supervised ensemble method presents a robust solution for hyperspectral image labelling and classification, with applications in environmental monitoring and remote sensing.

Lee et al., in contribution 16, tackle motion blur in images captured by thermal and photon detectors. They propose a method to synthesize blurry images from sharp ones by analyzing thermal detector mechanisms. Their novel blur kernel rendering method integrates motion blur models with an inertial sensor in the thermal image domain. Evaluation of its accuracy is conducted through thermal image deblurring tasks using a synthetic blurry image dataset constructed from acquired thermal images, the first to include ground-truth images in this domain. Through qualitative and quantitative experiments, the authors demonstrate the superiority of their method over existing techniques. In summary, the paper analyzes differences between thermal and photon detectors, developing a novel motion blur model for thermal images and an effective blur kernel rendering method, validated through rigorous experimentation.

Xiang et al., in contribution 17, introduced a novel approach for multi-sensor data fusion, crucial for information-aware systems with diverse sensory devices. Their method integrates the cloud model and an enhanced evidence theory to handle conflicting and ambiguous data. Quantitative data is converted into qualitative form using the cloud model to construct basic probability assignments (BPA) for each data source’s evidence. To resolve conflicts, similarity measures like Jousselme distance, cosine similarity, and Jaccard coefficient are combined to assess evidence similarity, while Hellinger distance calculates evidence credibility. Fusion is performed using Dempster’s rule. Experimental results show superior convergence and precision, achieving up to 100% confidence in correct propositions. Applied to early indoor fire detection, the method enhances accuracy by 0.9–6.4% and reduces false alarm rates by 0.7–10.2% compared to traditional methods, validating its effectiveness. Overall, this strategy offers a robust solution for managing conflicting and ambiguous data in information-aware systems, with promising applications across various multi-sensor acquisition systems. Future research should explore its applicability across different systems and integrate homogeneous and heterogeneous data fusion algorithms to further enhance accuracy.

Tropea et al., in contribution 18, introduced an automatic recognition system developed under the SILPI project, aiming to classify stones from quarries in Calabria, Southern Italy. Their two-stage hybrid approach combines Convolutional Neural Networks (CNNs) for feature extraction with Machine Learning (ML) for classification. Transfer Learning (TL) is explored to enhance CNN performance, using pre-trained networks from ImageNet. The system achieves impressive results in predicting stone classes, excelling in image recognition tasks. While granite typologies posed challenges, the hybrid model effectively integrates DL for feature extraction and classical ML algorithms for classification. The ResNet50 CNN model paired with a k-Nearest Neighbors (kNN) classifier emerges as the most promising combination, offering high accuracy, efficient CNN parameter usage, and rapid inference times. Overall, the approach demonstrates the potential for creating user-friendly tools applicable across various fields, including archaeometry, diagnostics, and materials sciences, even for users lacking geological expertise.

Rogers et al., in contribution 19, assessed RF measurement accuracy using Kubios HRV Premium software alongside consumer-grade hardware: Movesense Medical sensor single-channel ECG (MS ECG) and Polar H10 HR monitor. GE, RR intervals (from H10), and continuous ECG (from MS ECG) were collected from 21 participants during cycling exercises. Results showed strong correlations between reference GE and both H10 and MS ECG-derived RF. Median values differed statistically but were clinically negligible for H10 (about 1 breaths/min) and minimal for MS ECG (about 0.1 breaths/min). ECG-based RF measurement with MS ECG exhibited reduced bias and narrower limits of agreement than H10. The study concludes that MS ECG with Kubios HRV Premium software closely tracked reference RF during exercise, suggesting practical utility for endurance exercise. Additionally, the ECG-centric system outperformed RR interval-derived RF estimation, accurately capturing RF patterns during exercise ramps. Future studies should explore these findings across different exercise types and assess artifact and noise impact.

Benitez-Garcia et al., in contribution 20, introduced a new material translation method using Neural Style Transfer (NST). NST traditionally relies on reference image quality, which may not yield optimal results. To overcome this, their method incorporates automatic style image retrieval, selecting the ideal reference based on semantic similarity and distinctive material characteristics. Excluding style information during retrieval significantly enhances synthesized results. The method combines real-time material segmentation with NST to selectively transfer retrieved style image material to segmented object areas. Evaluation with different NST methods shows effectiveness, validated through human perceptual study indicating synthesized stone, wood, and metal images are perceived as real, surpassing photographs. Applications include creating alternate reality scenarios for users to experience environments with subtly modified objects. Future work should focus on synthesizing more materials and developing real-time material translation applications.

Dziech et al., in contribution 21, introduced a novel data-embedding technique based on the Periodic Haar Piecewise-Linear (PHL) transform. They explain the theoretical basis of the PHL transform and propose a watermarking method that embeds hidden information in the luminance channel of the original image using coefficients with low values. The method’s effectiveness is assessed by measuring the visual quality and bit error rate (BER) of watermarked images with different embedded information lengths. Additionally, a method for detecting image manipulation is presented. The technique shows promise for applications in digital signal and image processing, particularly in scenarios requiring high imperceptibility, low BER, and robust information security, such as medical image processing. The proposed method offers a high capacity for hidden information while minimizing image distortion, making it suitable for multimedia systems and services, especially in medical applications. Further research should focus on enhancing the method’s robustness against various attacks and exploring its potential applications across different domains.

The task of temporal action detection (TAD) in untrimmed videos is vital across various applications, predicting temporal boundaries and action class labels within videos. Current methods often use stacked convolutional blocks to capture long temporal structures but struggle with redundant information between frames and varying action durations. To tackle these issues, He et al., in contribution 22, propose a non-local temporal difference network with three key modules: chunk convolution, multiple temporal coordination (MTC), and temporal difference (TD). The CC module divides input sequences into chunks, extracting features from distant frames simultaneously. The MTC module aggregates multiscale temporal features without extra parameters, while the TD module enhances motion and boundary features with temporal attention weights. This approach achieves state-of-the-art results on ActivityNet-v1.3 and THUMOS-14 datasets, effectively capturing long-range temporal structures and enhancing TAD accuracy. Discussions underscore TAD challenges and the importance of efficient network design in modelling complex temporal relationships while considering video characteristics like varying action durations and information redundancy.

Conventional methods for repairing old photo damage are often slow and inefficient, relying on manual or semi-automatic processes that involve laborious marking of damaged areas. Fully automatic repair methods lack control over damage detection, posing risks to preserving historical photos. To overcome these challenges, Kuo et al. propose a deep learning-based architecture in contribution 23 to automatically detect damaged areas in old photos. The model accurately marks damaged regions, reducing damage marking time to less than 0.01 s per photo. By eliminating manual marking, the method enhances efficiency and preserves photo integrity. The use of residual dense block modules improves detection accuracy, ensuring preservation of both damaged and undamaged areas without distortion. Overall, this method provides a more efficient and precise approach to old photo restoration than existing end-to-end methods.

The study by Christou et al., in contribution 24, explores the influence of window size on EEG signal classification for diagnosing epilepsy. Automated analysis using machine learning is essential due to the complexity of EEG waveforms and the sporadic occurrence of epileptic characteristics. Employing various classifiers, including neural networks and k-nearest neighbour, EEG data from the University of Bonn dataset are analyzed with different window lengths. Results reveal that larger window sizes, approximately 21 s, notably enhance classification accuracy across tested methods. Given epilepsy’s significant impact, accurate and automated detection methods are crucial. The study underscores the importance of window size in EEG signal analysis and recommends epochs of 20–21 s for optimal classification performance.

Sebastião et al., in contribution 25 investigate pain perception by analyzing physiological responses, aiming to complement self-reporting methods in pain assessment. They recorded various physiological signals, such as ECG, EMG, EDA, and BP, during a pain-inducing protocol. Results demonstrated significant changes in physiological parameters during painful periods compared to non-painful ones, including increased heart rate and decreased PNS influence in ECG data, heightened muscle activity in EMG, and increased SNS activity in EDA. A novel data collection protocol enabled comprehensive analysis of ANS reactivity across body systems. The study highlights the importance of deeper physiological evaluation for understanding pain effects and suggests future research on multimodal classification for more reliable pain measurements. Limitations include the brief recording duration, emphasizing the need for longer protocols to explore SNS influences on the cardiovascular system further.

Wang et al., in contribution 26, introduced a denoising method tailored for partial discharge (PD) signals in mining cables. The method employs genetic algorithm optimization of variational mode decomposition (VMD) and wavelet thresholding to enhance the signal-to-noise ratio (SNR) by effectively separating PD signals from interference. Initially, the genetic algorithm optimizes VMD parameters such as the number of modal components (K) and quadratic penalty factor (α). Subsequently, VMD decomposition generates intrinsic mode functions (IMF), followed by wavelet threshold denoising of each IMF. The denoised IMF are then reconstructed to obtain the cleaned PD signal. Simulation and experimental verification confirm the method’s feasibility and efficacy, highlighting the optimized VMD parameters’ role in enhancing denoising performance and the synergy between VMD and wavelet thresholding for noise reduction without compromising transient processes. The method shows superior denoising ability, especially for PD signals with lower SNR, making it promising for PD monitoring in mining cables.

Shokouhyan et al., in contribution 27, highlight the significance of neuro-mechanical time delays in sensorimotor control, particularly in individuals with spinal cord injuries (SCI), impacting stabilization efficiency and system stability. Estimating these delays in SCI patients is crucial for designing effective rehabilitation exercises and assistive technologies. The study aims to estimate muscle onset activation in SCI individuals using four strategies on electromyography data. Results show that the total kinetic energy operator technique effectively reduces artifacts compared to classical filtering, while time-frequency techniques estimate longer delays due to lower frequency movement during seated balance. These estimated delays can inform sensory-motor control models and aid in designing tailored exercises and technologies for SCI rehabilitation.

As can be seen form the summaries presented above, 9 of the published works can be classified in the field of image and multidimensional signal processing and applications, 11 in the field of signal processing and applications, and 7 in the field of data processing and applications. Additionally, it is important to highlight the fact that 11 of these works have direct applications in health related areas.

Last, but not least, I want to extend my personal gratitude to all the authors and reviewers who have contributed to this Special Issue. The authors deserve recognition for their innovative ideas and solutions, while the reviewers deserve appreciation for dedicating their time and offering valuable improvement suggestions. Their outstanding efforts have enabled Sensors journal to showcase novel and compelling contributions in the realm of “Data, Signal, and Image Processing and Applications in Sensors II”. I final word of thanks goes to the Sensors journal’s staff for their continuous support and suggestions. Thank you to each and every one of you!

